# Air Pollution: Ship Sulfate an Unexpected Heavyweight

**Published:** 2008-11

**Authors:** Adrian Burton

A technique that can identify the source of sulfate (SO_4_) produced during combustion—or primary SO_4_—has shown ships may be responsible for a large proportion of this pollutant in the fine particulate matter suspended in Southern California’s coastal air. This is a much higher percentage than regulators thought likely and—given that fine particles are harmful to human health—much more than anyone would like.

Ships commonly burn sulfur-rich “bunker oil,” but until now, it has been difficult to tease out the contributions to air pollution of this and other transport fuels or to distinguish anthropogenic components of sulfate pollution from the natural sulfate aerosols in the tropospheric background. This new research suggests that ships burning bunker oil are responsible for a significant fraction of sulfate particles in coastal areas.

“Our technique works on the principle of oxygen isotope ‘fingerprinting,’” explains research leader Mark Thiemens, dean of the Division of Physical Sciences at the University of California, San Diego (UCSD). “By knowing these fingerprints, we can work out how much primary sulfate comes from each source in air samples.” The research appears in the 2 September 2008 issue of the *Proceedings of the National Academy of Sciences*.

The researchers collected samples of smoke from the stack of a ship burning bunker oil, from the end of the Scripps Pier in La Jolla, California, and from an unpolluted coastal area off Northern California. They chemically isolated the samples’ SO_4_ content as silver sulfate. This was then combusted to release its oxygen, which was analyzed with a mass spectrometer to determine its ratio of δ^18^O, δ^17^O, and Δ^17^O isotopes—thus providing an isotopic fingerprint of the SO_4_.

Brad Collins, a chemist with the National Toxicology Program, explains that δ^17^O refers to the ratio of the ^16^O to ^17^O isotopes in a sample, whereas δ^18^O refers to the ratio of ^16^O to ^18^O isotopes. The Δ^17^O value refers to how much the δ^17^O values deviate from what would be expected on the basis of mass-dependent fractionation of the isotopes. “For example,” Collins says, “^16^O water molecules vaporize more easily than ^17^O water molecules because they are lighter. So based on the mass alone, you would expect rain [being condensed water vapor] to be deficient in ^17^O water molecules by a certain amount. The deviation from what you expect and what you actually find is Δ^17^O. You can use these ratios to look for anthropogenic sources of oxygen-containing substances.”

“By comparing the isotope fingerprints of sulfate in pristine coastal air, the stack smoke sulfate . . . and [pier] air samples, we showed that the ship smoke was making a large contribution to the fine particle sulfate in [those pier air samples],” says first author Gerardo Dominguez, a postdoctoral fellow in the UCSD Department of Chemistry and Biochemistry. In fact, on some days this contribution reached 44%.

To date, however, SO_4_ has been largely ignored in environmental impact assessments of ship smoke. “This may have to change,” says Dominguez, who explains that these fine particles build up over time and can travel long distances. If the contributions observed in this study are typical, he says, primary SO_4_ from ships could account for 4–25% of the 15-μg/m^3^ annual maximum fine particle exposure limit set by the U.S. Environmental Protection Agency.

“Epidemiological studies have associated particulate matter with increased risk of respiratory illness and cardiopulmonary mortality,” says Spyros Pandis, a research professor of chemical engineering at Carnegie Mellon University. “To the best of our knowledge sulfate particle effects are similar to those of [nonsulfate] particles. This is the reason that regulations in both the United States and Europe target particulate mass and not the mass of, say, organic particles or sulfate.”

“The fingerprinting technique is excellent and allows better discrimination of the natural and anthropogenic sources of the various aerosols in the marine atmosphere,” adds Geoff Millward, a professor of marine chemistry at Plymouth University, United Kingdom. “The technique might have some application in the policing of emissions.”

Indeed, lawmakers are requiring vessels to switch from bunker oil to lower-sulfur marine distillate fuel on the basis of evidence such as findings published by James J. Corbett and colleagues in the 15 December 2007 issue of *Environmental Science & Technology*, which suggest that total smokestack emissions from ships may be responsible for 60,000 cardiopulmonary and lung cancer deaths annually worldwide. By 2012, oceangoing vessels calling on California ports must switch to marine distillate fuel with a sulfur content of 0.1% within 24 nautical miles of the California coastline, says Michael Robinson-Dorn, director of the Kathy and Steve Berman Environmental Law Clinic at the University of Washington (currently the sulfur content in bunker fuel used in California averages about 2.4%). Similarly, European Union Directive 2005/33/EC mandates that inland waterway vessels and docked ships must use 0.1% sulfur marine distillate fuel by 2010.

## Figures and Tables

**Figure:**
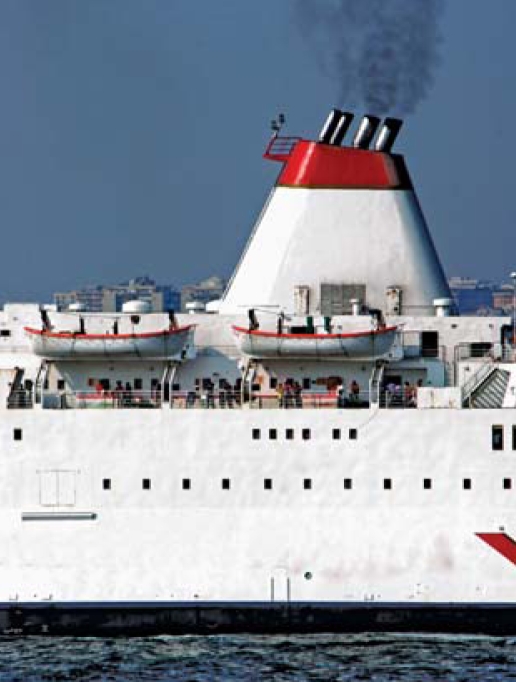
SO_4_ in ship smoke has largely flown under the radar—until now.

